# Dissecting industrial fermentations of fine flavour cocoa through metagenomic analysis

**DOI:** 10.1038/s41598-021-88048-3

**Published:** 2021-04-21

**Authors:** Miguel Fernández-Niño, María Juliana Rodríguez-Cubillos, Fabio Herrera-Rocha, Juan Manuel Anzola, Martha Lucia Cepeda-Hernández, Jenny Lorena Aguirre Mejía, María José Chica, Héctor Hugo Olarte, Claudia Rodríguez-López, Dayana Calderón, Adan Ramírez-Rojas, Patricia Del Portillo, Silvia Restrepo, Andrés Fernando González Barrios

**Affiliations:** 1 Grupo de Diseño de Productos Y Procesos (GDPP), Departamento de Ingeniería Química Y de Alimentos, Universidad de Los Andes, 111711 Bogotá, Colombia; 2 Department of Bioorganic Chemistry, Leibniz-Institute of Plant Biochemistry, Weinberg 3, 06120 Halle, Germany; 3 Laboratorio de Micología Y Fitopatología (LAMFU), Departamento de Ingeniería Química Y de Alimentos, Universidad de Los Andes, 111711 Bogotá, Colombia; 4 Grupo de Bioinformática Y Biología de Sistemas, Corporación CorpoGen, Bogotá, Colombia; 5 Universidad Central, Bogotá, Colombia; 6 Facultad de Ciencias, Universidad de Los Andes, 111711 Bogotá, Colombia; 7 Grupo de Biotecnología Molecular, Corporación CorpoGen, Bogotá, Colombia; 8 CASALUKER S.A., Bogotá, Colombia

**Keywords:** Biotechnology, Industrial microbiology, Environmental microbiology, Food microbiology

## Abstract

The global demand for fine-flavour cocoa has increased worldwide during the last years. Fine-flavour cocoa offers exceptional quality and unique fruity and floral flavour attributes of high demand by the world's elite chocolatiers. Several studies have highlighted the relevance of cocoa fermentation to produce such attributes. Nevertheless, little is known regarding the microbial interactions and biochemistry that lead to the production of these attributes on farms of industrial relevance, where traditional fermentation methods have been pre-standardized and scaled up. In this study, we have used metagenomic approaches to dissect on-farm industrial fermentations of fine-flavour cocoa. Our results revealed the presence of a shared core of nine dominant microorganisms (i.e. *Limosilactobacillus fermentum*, *Saccharomyces cerevisiae*, *Pestalotiopsis rhododendri*, *Acetobacter aceti* group, *Bacillus subtilis* group, *Weissella ghanensis* group, *Lactobacillus_uc*, *Malassezia restricta* and *Malassezia globosa*) between two farms located at completely different agro-ecological zones. Moreover, a community metabolic model was reconstructed and proposed as a tool to further elucidate the interactions among microorganisms and flavour biochemistry. Our work is the first to reveal a core of microorganisms shared among industrial farms, which is an essential step to process engineering aimed to design starter cultures, reducing fermentation times, and controlling the expression of undesirable phenotypes.

## Introduction

Cocoa consumption has increased worldwide during the last years in response to its use as a high-quality ingredient in food and nutraceutical products^1^. Currently, the global market of cocoa is segmented into two different categories: bulk cocoa (95% of the global production) and fine-flavour cocoa (the remaining 5%)^2^. Fine-flavour cocoa refers to cocoa of exceptional quality and unique flavour attributes (e.g. fruity notes), a highly appreciated attribute by the world's elite chocolatiers. According to International Cocoa Organization (ICCO), 95% of the Colombian cocoa production is recognized as fine-flavour and its production is expected to reach 246,000 t by 2021 in response to the Cocoa development 10-year plan 2012–2021^3^. Moreover, this crop has been in the spotlight for replacing illegal crops (Colombia’s Crop Substitution Program: *From Coca to Cocoa*) that aims at contributing to the economic development of farmers in high-risk areas, as fine-flavour cocoa offers a new range of unique fruity and floral tastes reaching high-value markets and a premium price^4^.

High-quality chocolate is traditionally obtained through extensive processing including on-farm spontaneous fermentation, drying, and roasting, which together play an essential role in the production of compounds affecting quality and taste^5^. More than 600 compounds including alcohols, aldehydes, ketones, esters, pyrazines, acids, peptides, and phenols have been reported to be involved in cocoa flavour as described by Ziegleder^6^ and reviewed by Aprotosoaie et al.^2^. Several studies have shown that these compounds can be produced directly during fermentation and kept stable during subsequent roasting stages^7–10^, while some of them experienced changes during drying and roasting^11^. Nevertheless, little is known regarding the microbial interactions and the biochemistry that lead to the production of such compounds during spontaneous cocoa fermentations. It has been described that spontaneous cocoa fermentations involves microorganism such as yeasts, lactic acid bacteria (LAB), acetic acid bacteria (AAB), filamentous fungi and spoilage bacteria^12–17^, which may come from the environment (e.g. air and insects), collecting tools, farmers hands, plant microbiota and the selected fermentation system (e.g. wooden boxes). On the other hand, spontaneous cocoa fermentations also depend on environmental parameters such as humidity, altitude, wind, rain, and temperature, which vary from one region to another and among different fermentation practices^16,17^.. Despite some traditional fermentation methodologies have been successfully standardized at large scales, cocoa bean fermentation remains a spontaneous/uncontrolled process. Thus, the identification of a core of dominant microorganisms at different locations is an essential step to design started cultures in controlled fermentations.

Up to now, several studies have focused on the identification of microorganisms during bulk cocoa fermentations^17–22^. Comprehensive studies regarding the fermentation of fine-flavour cocoa beans are rare^9,23^ and this field is still in its infancy. Very recently, the effect of different on-farm traditional fermentation protocols and environmental conditions in the composition of microbial communities have been studied in two different agro-ecological zones in Colombia^23^.The authors evaluated the effect of dissimilar fermentation protocols, climate, and other parameters on the microbial composition over time. The fermentation protocols at their selected farms were heterogeneous and even changed with the same farm at different seasons. Unfortunately, the authors do not state which clones or clones’ mixtures have been used for each fermentation and what could be their impact on microbial assembly. In this study, we are going one step further by using metagenomic approaches to dissect fermentations of fine-flavour cocoa on farms of industrial relevance (i.e. annual production exceeding 280 tons), where traditional fermentation methods have been scaled up using pre-designed mixtures of clones and reproducible fermentations methodologies. Instead of focusing on spontaneous fermentations on farms with dissimilar protocols, our goal was to identify a core of microorganism between industrial farms, where the same mixture of clones and fermentation methodologies have been used for many years to guarantee reproducibility. Thus, we have used a pre-standardized mixture of fine flavour clones, which is a standard used by the company to produce its origin chocolate. This approach allowed the identification and characterization of a common microorganism’s core shared between two different large-scale producing farms located at two completely different agro-ecological zones in Colombia. Dominant species and microbial groups have been also identified and analyzed regarding the product quality at each farm. Besides, we have reconstructed a genome-scale metabolic model departing from selected species of this core that were integrated to obtain a community metabolic model for the fermentation of fine-flavour cocoa.

## Results and discussion

### A platform to study on-farm industrial fermentations of fine-flavour cocoa

Cocoa bean fermentation has been extensively reported as a crucial step to produce flavour attributes and bioactive compounds of economical relevance in cocoa products^5,6,8,10,11,24^. Unlike other fermented foods, cocoa fermentation is an unstandardized/spontaneous process with a large variability all over the world. Currently, there is no universal standard methodology to perform cocoa fermentation and only a few international companies have done a substantial effort to scale up and optimize traditional approaches. Thus, a current challenge in cocoa research is the development of a large-scale standardized methodology that can be transferred to farms of industrial relevance. This is an important step to bring on-farm fermentations to industrial production scales, where controlled and reproducible protocols can be used to guarantee a standard product quality.

**Figure 1:**
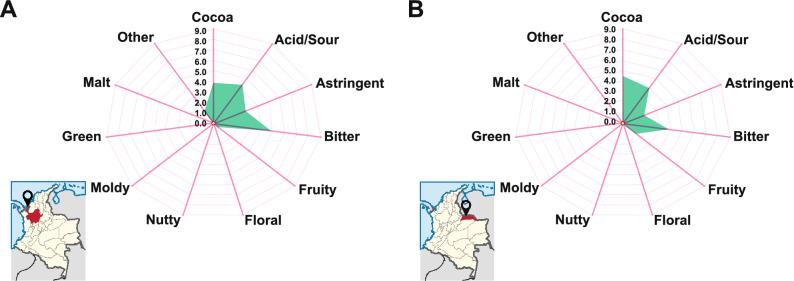
Sensory profiles of cocoa liquor samples produced in Colombia by CASALUKER S.A., from a mixture of commercial clones of fine-flavour cocoa beans from Necoclí (**A**) and Arauca (**B**) farms (for more details on selected clones see experimental section). The flavour intensity increases from the centre to the perimeter. Data were obtained through a sensory analysis performed by a trained tasting panel of 6 members. Three replicates were performed. Mean values are shown.

In this work, a platform to study on-farm industrial fermentations of fine-flavour cocoa (i.e. annual production exceeding 280 t) has been established as explained in the experimental section. Thus, two farms of industrial relevance were selected (i.e. Necoclí farm and Arauca farm). The selected farms are located at two completely different agro-ecological zones in Colombia (Fig. 1) and separated by the Andes Mountains (approximately 610 km from each other). The Necoclí farm was founded in 2010 by CASALUKER S.A and is situated on the Caribbean coast (8°28′48.24″N 76°40′59.86″W). It represents the largest fine-flavour cocoa crop in Colombia (550 ha) with an annual production of 280 t. Cocoa liquor from Necoclí farm is known to present an aroma with sour tones, light toasted notes, flavour with pronounced acidity (acetic and citric), a medium balance between cocoa and bitter, medium–low astringency, delicate sweet note (caramel and fruity), and slightly woody tones. In contrast, liquor from Arauca farm (7°01′01″N 71°25′01″W-COMPROCAR collection centre) is recognized for its unique fruity aroma, being the winner of several international chocolate awards during the last decade (e.g. *Salon du Chocolat de París 2010–2011*). It is known to present an aroma with sour and sweet notes, flavour with delicate sweet tones, and a medium balance between cocoa and bitter, lightly citric and fruity notes, low astringency, mild bitterness, and pronounced acidity. These sensory characteristics are frequently observed on the cocoa liquor produced at both farms where fermentation methodologies and clones’ mixtures are standardized. Sensory evaluations are regularly performed several times a year by the company, always producing the same distinguished profile. In this study, we have obtained the same sensory profiles by using the same methodology and clone’s mixture standardized at each farm described in the experimental section (Fig. 1).

In this study, two wooden large-scale box fermentations were arranged at each farm following the standard methodology used for the company and described in the experimental section. Fermentations at both farms were also studied by recording environmental parameters such as the temperature of the cocoa beans mass and pH (two frequently used parameters to verify proper fermentation at industrial farms). Thus, the temperature profile over fermentation was recorded and showed to be similar between the studied farms (Supplementary Fig. S1). The maximum temperature is always expected in the middle of the fermentation and it is used as a parameter to determine a good quality fermentations^25^. An increase in temperature during fermentation is frequently associated with ethanol production as an exothermic process^25–27^. The temperature of fermentation mass can vary from 25 to 55 °C depending on several parameters such as aeration, selected clones, fermentation methodology, room temperature, humidity, and other environmental parameters^25–27^. Similarly, the pH of the cocoa beans mass dropped from 6.5 to 6.6 during the first day to 5–5.6 at the end of fermentation at both farms (data not shown) as frequently reported in cocoa fermentation in response to the production of weak acids by acetic and lactic acid bacteria^28^.

### Interesting variations in diversity and abundance of relevant groups of microorganisms over fermentation were found at the studied farms

In this work, we aimed to dissect industrial large-scale fine-flavour cocoa fermentation by using metagenomic approaches. Consequently, the variations of microbial diversity and composition in fermentations at the selected farms were determined as detailed explained in the experimental section.

**Figure 2:**
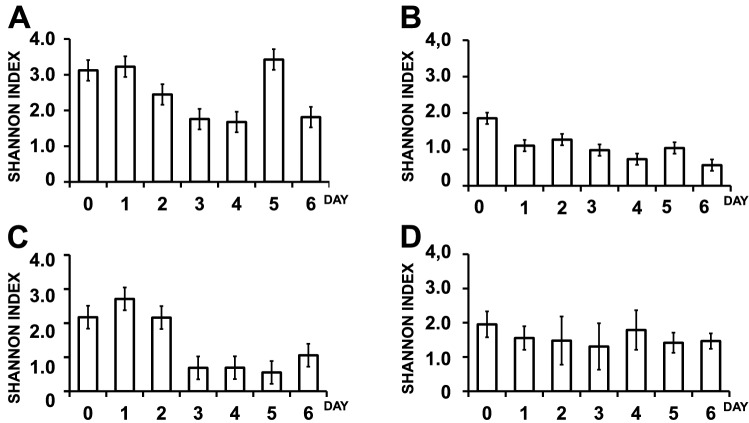
Changes in the Shannon Diversity Index during the fermentation of fine flavour cocoa beans at Necoclí (**A**,**B**) and Arauca (**C**,**D**) farms. Data is shown for bacteria (**A**,**C**) and fungi (**B**,**D**). Beans samples were collected every 24 h over fermentation as described in the methods section.

Initially, the changes in the Shannon Diversity Index for bacteria and fungi during the fermentation of fine flavour cocoa beans at Necoclí and Arauca farms were recorded (Fig. 2). Our data revealed a decrease in bacterial and fungal diversity during fermentation at both farms, which is concomitant with the reported production of typical metabolites affecting microbial growth such as ethanol and weak acids^29^ and the observed changes in beans mass temperature (Supplementary Fig. S1). This may suggest the creation of a restrictive microenvironment during spontaneous industrial fermentations at both farms. Interestingly, a higher diversity of bacteria was always observed at Necoclí farm as compared to Arauca farms all over the tested replicates (Fig. 2A–C), while fungal diversity was similar between the studied farms (Fig. 2B–D).

**Figure 3:**
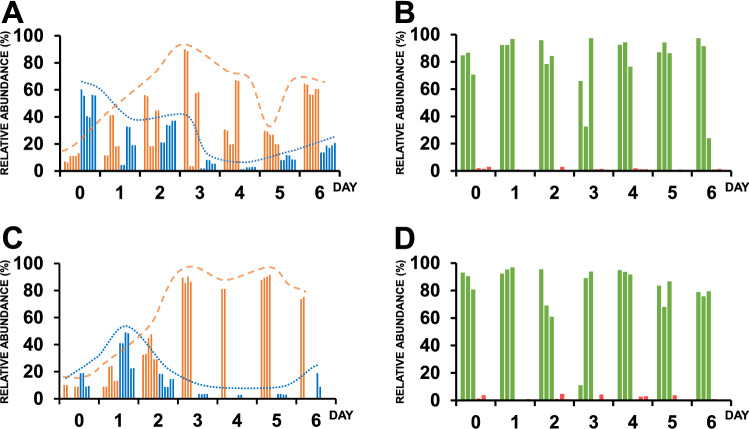
Relative abundance of selected microorganism groups sharing common metabolic and physiological characteristics during the fermentation of fine flavour cocoa beans. Schematic representation of the relative abundance of Acetic Acid Bacteria (orange bars) and Lactic Acid Bacteria (blue bars) and yeasts (green bars) and molds (red bars) identified at Necoclí farm (**A**,**B**: Necoclí farm, **C**,**D**: Arauca farm). Orange and blue lines were used to illustrate the behaviour of AAB and LAB over the fermentation, respectively. Each bar represents a replicate. At least two replicates per day were analysed.

To further dissect the microbial composition over fermentation, the relative abundance of selected groups of microorganisms sharing common metabolic characteristics was evaluated every day at the selected farms as mentioned in the experimental section. Selected microorganisms sharing metabolic traits were subsequently clustered in four groups: Lactic Acid Bacteria (LAB), Acetic Acid Bacteria (AAB), Yeasts, and Molds. Their relative abundance was recorded every 24 h until the end of fermentation (Fig. 3). Changes in the relative abundance of LAB and AAB over fine-flavour cocoa fermentations were observed all over the tested replicates at both farms. LABs are recognized as a crucial group of microorganisms in cocoa fermentations^30^. Our data revealed a higher abundance of these microorganisms at both farms during the first 3 days of fermentation, as reported in the literature^28,29^. The ability of LAB to transform pulp’s fermentable sugars and citric acid into lactic acid, mannitol, and pyruvate has been previously described^31^. Such metabolites have been reported to be essential for the release of sour taste during fermentation and spoilage control^2,5,30,32,33^. Indeed, the lactic acid produced by this group of bacteria is known to be an essential carbon source for the second group of relevant microorganisms during cocoa fermentation: the AAB^30^. Like LAB, AAB species have been previously reported to play an important role in flavour development^5,34^ and by affecting the microbial community structure ^33,35^. Moreover, their ability to convert ethanol into acetic acid is very important to allow the release of enzymes from the dead seeds, which are crucial to producing flavour precursors such as hydrophilic peptides, hydrophobic amino acids, polyphenols, and reducing sugars^2,5,8,9^. At both farms, the relative abundance of AAB was observed to increase over fermentation (Fig. 3) as previously reported^28,29^.

The relative abundance of yeasts, a key group of microorganisms on cocoa fermentation, was also studied at the selected farms (Fig. 3B–D). Yeast species are known to play an important role to transform pulp’s fermentable sugars into ethanol during the first days of fermentation, where an anaerobic environment predominates^12,28,33^. This group of microorganisms has been also associated with the hydrolytic dissolution of pectin, which is an important step for the release of organic acids, aldehydes, and esters associated with flavour^30^. Interestingly, yeast flavour-active compounds have been widely associated with aroma in spirits, beers, and other fermented products^36^. Further metabolomic analysis will be required to highlight their relevance on flavour development during fine-flavour cocoa fermentations. The relative abundance of yeasts over the whole fermentation was similar at both farms (Fig. 3B–D). Our results are different from the observed on bulk cocoa fermentations^28,30^, where yeasts abundance is reduced during the last days of fermentation possibly due to the accumulation of weak acids ^28,30^. In this study, yeast abundance was high during the whole fermentation process at both farms. A possible explanation for the observed behaviour in this study relies on the fact that yeast strains display high variability in their response to weak acids, thus their abundance could not be strongly affected by acid concentration^37^. However, it is important to mention that no formal studies have compared the weak acid concentrations at different industrial and local farms. Moreover, the differences in the response to weak acids between yeast strains from different farms still have to be elucidated and additional studies will be required to further dissect this behaviour.

Finally, the changes in the relative abundance of mycotoxins-producing molds were evaluated at the selected farms (Fig. 3). Their relative abundance was always low during the whole fermentation at both farms.

Although our data allowed us to identify the variations on key groups of microorganisms during fermentation, we wanted to go one step further by identifying dominant microbial species at the selected farms, which is a crucial step for the design of starter cultures and process engineering. However, the design of starter cultures for cocoa bean fermentation will require to first achieve several challenges as recently reviewed by Figueroa-Hernández et al.^38^. For instance, it will be first necessary to standardize fermentations according to their location, the mixture of selected clones, and additional omics data.

### A core of microorganisms shared between industrial farms at different agro-ecological zones was identified

**Figure 4:**
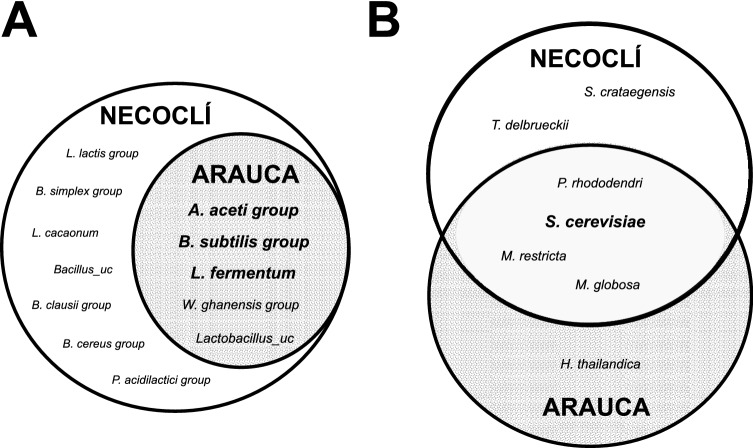
Schematic representation of dominant species observed during the fermentation of fine-flavour cocoa beans at Necoclí and Arauca farms. Dominant species were defined as species detected all over the replicates and showing a relative abundance higher than 1% from the total number of bacterial or fungal species. Data is shown for bacterial (**A**) and fungal species (**B**). Intersections on Venn Diagrams represent dominant species identified at both farms (here referred to as microorganism’s core). Bold highlighted names indicate the species selected for genome-scale metabolic model reconstruction that was later integrated to produce a community metabolic model for the fermentation of fine-flavour cocoa.

**Figure 5:**
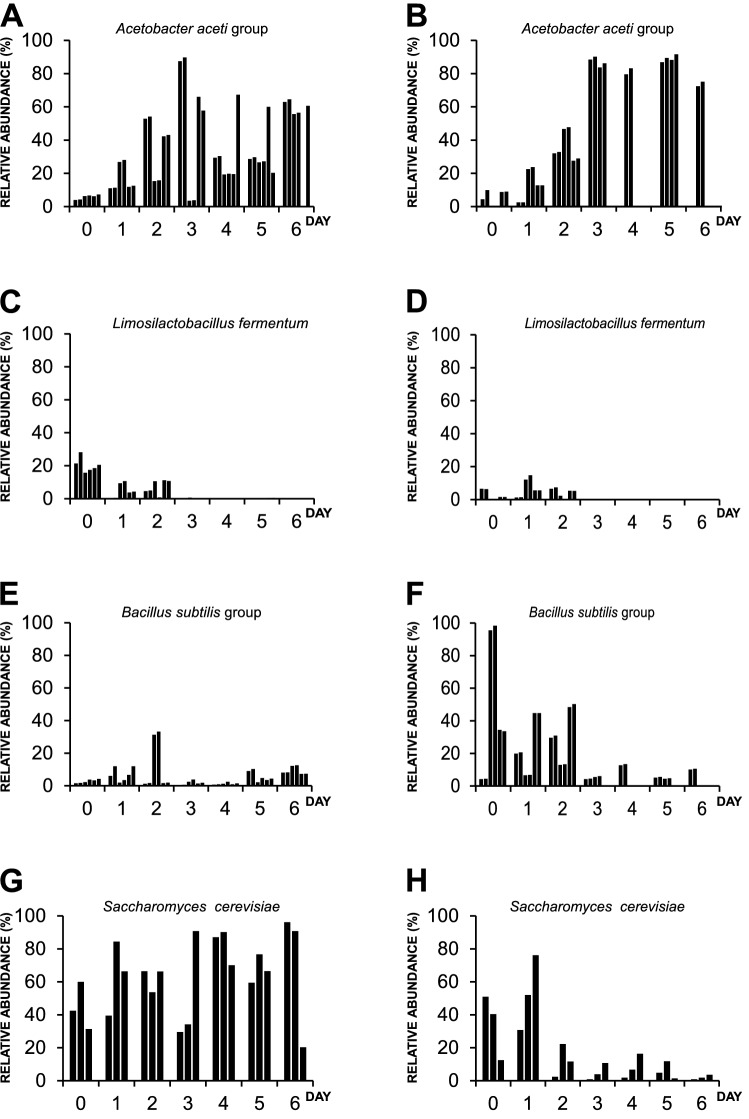
Relative abundance of dominant species of bacteria and fungi from the microorganism’s core during the fermentation of fine flavour cocoa beans at Necoclí (**A**,**C**,**E** and **G**) and Arauca (**B**,**D**,**F** and **H**) farms. Data are shown only for species included in the metabolic model (for remaining species included in the core see Supplementary Fig. S3). Dominant species were defined as species detected all over the replicates and showing a relative abundance higher than 1% from the total number of bacterial or fungal species. Each bar represents a replicate. At least two replicates per day have been analysed.

Bacterial and fungal species were identified every day of fermentation as explained in the experimental section. Our data revealed a core of nine dominant microbial species shared between the studied farms (Fig. 4). Dominant microbial species were defined as those fulfilling the following criteria: (I) species detected all over the tested replicates, and (II) species with relative abundance higher than 1% of the total number of bacterial or fungal species. Thus, the relative abundance of selected dominant bacterial and fungal species was recorded every day of fermentation at each farm (Fig. 5 and Supplementary Fig. S2).

A total of 18 dominant microbial species were found at Necoclí farm, while 10 dominant species were found at Arauca farm (Fig. 4). Interestingly, all dominant bacterial species identified at Arauca farm (i.e. *Acetobacter aceti* group, *Limosilactobacillus fermentum*, *Bacillus subtilis* group, *Weissella ghanensis* group, and *Lactobacillus_uc*) were also found to dominate fermentation at Necoclí farm (Fig. 4A). Only four fungal dominant species were shared between the studied farms (i.e. *Pestalotiopsis rhododendri*, *Saccharomyces cerevisiae*, *Malassezia restricta,* and *Malassezia globosa*) (Fig. 4B).

The *Acetobacter aceti* group was found to dominate fermentation at both farms as shown in Fig. 5 and Supplementary Fig. S3. The relative abundance of this group of acetic acid bacteria (AAB) increased over fermentation (Fig. 5A,B), which is a frequently reported behaviour^2,5,30^. Interestingly, the *Acetobacter aceti* group was also found to be present and dominate the whole fermentation (Fig. 5A,B). This could partially explain the distinctive sour taste of chocolate liquor produced from both farms (Fig. 1). The role of this group of AAB on the release of sour taste during fermentation and spoilage control has been previously described^2,5,30,32^. In fact, acetic acid is recognized as a crucial driver of flavour cocoa development^5,34^ and a potential microbial inhibitor^33,35^. A similar role in the control of microbial populations^33^ and sour taste^5^ has been attributed to the lactic acid bacterium *Limosilactobacillus fermentum*, which is recognized as a representative lactic acid bacterium (LAB) in spontaneous cocoa bean fermentations^39,40^. *Limosilactobacillus fermentum* was identified as a dominant species during the first days of fermentation at both farms as shown in Fig. 5C,D. The lactic acid produced by LAB is recognized as an essential carbon source for AAB^30^. Moreover, it contributes together with AAB to the production of weak acids involved in embryo death, thus allowing the release of enzymes (e.g. proteases, lipases) present in the seeds and several flavour precursors^2,5,8,9^. In this work, we also identified the lactic acid bacterium *Weissella ghanensis* recently reported on Ghanaian cocoa fermentations^41^ and colombian coffee fermentations^42^. *Weissella ghanensis* showed similar behaviour to *Limosilactobacillus fermentum* being dominant only during the first days of fermentation (Supplementary Fig. S2A,B). This species is reported for the first time in Colombian cocoa fermentations.

Interestingly, our data also revealed the relevance of the *Bacillus subtilis* group during the fermentation of fine flavour cocoa at both farms (Fig. 4). This species group has been previously identified on fermented protein foods such as shoyu and miso^43^. As shown in Fig. 5 and Supplementary Fig. S3, it was found to dominate all over the fermentation at both farms. In fact, it was the main dominant on day 0 at Arauca farm and its abundance was relatively higher as compared to Necoclí farm (Fig. 5E,F). Although the presence of the *Bacillus subtilis* group on cocoa fermentations has been early described in1986 by Schwan et al.^44^, its metabolic contribution to fermentation still has to be elucidated.

In contrast to the *Bacillus subtilis* group, the role of *Saccharomyces cerevisiae* on spontaneous fermentation has been extensively described^12,28,33^. This species has been identified as dominant at both studied farms (Figs. 4 and 5). As expected, its relative abundance was shown to decrease over fermentation at Arauca farm (Fig. 5H). This behaviour has been associated with the production of inhibitory weak acids during the last days of fermentation^28,29^. Surprisingly, the abundance of *Saccharomyces cerevisiae* at Necoclí farm was always higher throughout the fermentation step (Fig. 5H). This could be partially explained by the fact that a relatively lower abundance of AAB was observed at this farm as compared to Arauca farm (Fig. 3A–C). However, additional studies will be required to further dissect this particular behaviour, for instance, it is known that the tolerance to acetic acid is a strain-dependent parameter with a large degree of variation among *S. cerevisiae* strains^37^.

It is important to mention that other fungal species were also identified as part of the core of the dominant microorganism. For example, our data revealed the presence of *Pestalotiopsis rhododendri*^45^ as a dominant species at both farms. This is the first report of this phytopathogen being a dominant relevant species on spontaneous cocoa fermentation. Further studies will be required to elucidate the role of this and other phytopathogens on cocoa fermentation. Similarly, two species of the human-related genus *Malassezia*^46^ were identified as dominant at both farms (Fig. 4 and Supplementary Fig. S2). This is also the first report of *Malassezia globosa* and *Malassezia restricta* being dominant on cocoa fermentation. Interestingly, these species can utilize fatty acids as a carbon source, which could affect the lipids profile over fermentation. Nevertheless, further research will be required to test this hypothesis and elucidate their role on cocoa fermentations.

In this study, we have identified a core of nine dominant microorganisms shared between the studied farms. This is a viable tool that can be used to design and develop starter cultures. This is an important step to switch from spontaneous to controlled on-farm fermentations. Nevertheless, additional experiments will be required to increase the number of studied farms and to identify the potential of each nominated species using metabolomics analysis. Besides, we have identified species that are present only on a particular farm (Fig. 4). Such unique species could be used to further explore the microbial differences that could influence variation on the flavour profiles among different agro-ecological zones.

### The first community metabolic model for the fermentation of fine-flavour cocoa was reconstructed

A genome-scale metabolic community model was reconstructed to further dissect industrial fermentations of fine flavour cocoa. Hence, four dominant species were selected from the core of microorganisms shared between the studied farms: *S. cerevisiae, B. subtilis, L. fermentum and A. aceti* (Figs. 4 and 5). Three selection criteria were used to nominate these species: (I) to be present at both evaluated farms as shown in Fig. 4; (II) to represent a functional group of microorganisms involved in the production of typical cocoa fermentation metabolites (i.e. ethanol, acetic acid, and lactic acid) and (III) to dominate at several points through fermentation as shown in Supplementary Fig. S3.

To reconstruct a community metabolic model with these species, two previously reported metabolic models were initially selected: the iMM904 metabolic model for *S. cerevisiae*^47^ and the iYO844 metabolic model for *B. subtilis*^48^. The *S. cerevisiae* metabolic model iMM904 was selected as it is recognized as a model of preference to study central carbon metabolism and aminoacids biosynthesis^49^. It is a compartmentalized model composed of 1226 metabolites, 1577 reactions, and 905 genes.

Regarding the *B. subtilis* iYO844 metabolic model, it was selected as it represents the unique metabolic reconstruction reported at the BiGG database^50^ for this microorganism. It is composed of 990 metabolites, 1250 reactions, and 844 genes.

In the case of *L. fermentum* and *A. aceti*, de novo metabolic models had to be reconstructed in this study from their reported genomes. The *L. fermentum* str. IFO 3956 genome (GCF_000010145.1)^51^ and *A. aceti* str. TMW2.1153 genome (GCF_002005445.1)^52^ were then selected to reconstruct de novo metabolic models using the PATRIC database tools^53,54^. These genomes were selected as they were assembled as one contig, they were annotated into the PATRIC database, and their reconstruction resulted in a large number of reactions related to central carbon metabolism and amino acids biosynthesis. The resulting *L. fermentum* metabolic model is composed of 1238 metabolites, 1135 reactions, and 1030 genes. For *Acetobacter aceti*, the reconstructed metabolic model contains 1323 reactions, 1444 metabolites, and 1246 genes. Flux Balance Analysis (FBA)^55^ was then used to analyze the flow of metabolites into every reconstructed model, which converged to a solution of 29,053 gDW-1 h-1 for *L. fermentum* and 148,197 gDW-1 h-1 for *A. aceti*. Complete media was used as a standard to reconstruct each model as it contains all the required metabolites to fill the gaps. Future metabolomics analysis will be required to improve our model in terms of its biochemical composition.

**Figure 6:**
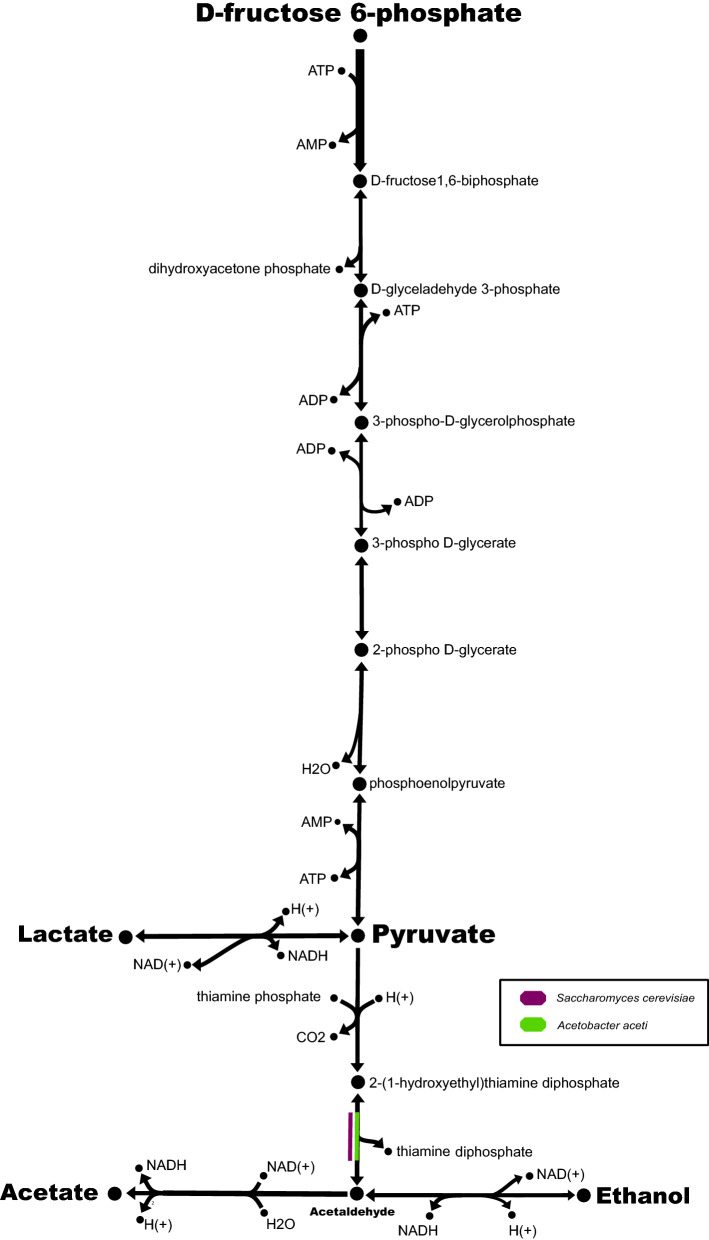
General metabolic network of the central carbon metabolism reconstructed at the community level. The genome-scale metabolic models of four dominant species shared between the studied farms (i.e. *S. cerevisiae*, *B. subtilis*, *L. fermentum* and *A. aceti*) were used to reconstruct a metabolic community model for fine flavour cocoa fermentation. Production pathways for lactate, ethanol, and acetate are shown (bold-highlighted). Black arrows represent metabolic reactions occurring in all the four species. Colours were used to indicate reactions that took place only on the indicated microorganism.

**Figure 7:**
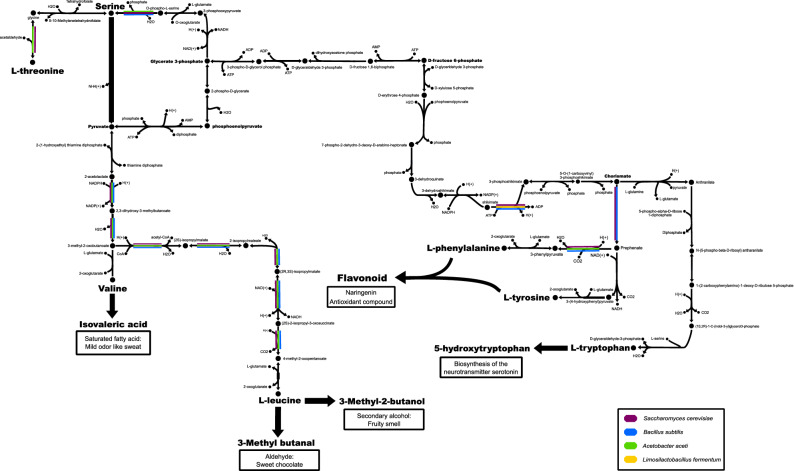
Network diagram of the amino acid synthesis pathway reconstructed at the community level. Production pathways for l-leucine, l-phenylalanine, l-tyrosine. l-tryptophan, valine and l-threonine are shown (bold-highlighted). These aminoacids have been recognized as crucial precursors of flavour/odour (e.g. 3-Methyl-2-butanol) and bioactive compounds (e.g. naringenin) in fine flavour cocoa. Black arrows represent metabolic reactions occurring in all the species of the community model (i.e. *S. cerevisiae*, *B. subilis*, *L. fermentum* and *A. aceti*). Colours were used to indicate reactions that took place only on the indicated species.

Once these four independent models were obtained, they were integrated into a unique genome-scale community metabolic model (Supplementary File S1) using the SteadyCom optimization framework^56^. This model contains 5.432 reactions and 4.885 metabolites, thus allowing the identification of reactions of relevance to produce typical cocoa fermentation metabolites (Fig. 6) and flavour precursors (Fig. 7). As shown in Fig. 7, the reported model represents an essential tool for the identification of species-dependent reactions occurring during fermentation. Interestingly, the production of L-leucine is a fundamental step to produce 3-Methyl-2-butanol and 3-Methylbutanal (two important metabolites previously related to fruity and sweet chocolate flavour in fine flavour cocoa products^2^). While l-phenylalanine and l-tyrosine are related to the production of naringenin, an important precursor of antioxidant compounds of high relevance in the food industry^57^.

Our work represents an integrative approximation to understand the metabolic interactions of dominant species during the fermentation of fine-flavour cocoa. Up to now, research has been mainly focused on the reconstruction of individual metabolic models for the typical species of cocoa fermentation^58,59^. Recently, mathematical models of cocoa bean fermentation have been constructed based on current microbiological and biochemical knowledge^28,29^. Here, we go one step further by integrating data from genome-scale metabolic reconstructions of several dominant species found at two different farms of industrial relevance. Thus, a genome-scale community metabolic model for industrial fermentations of fine-flavour cocoa has been reconstructed. This model could be used as a platform for the identification of several species-dependent reactions of relevance for process engineering/fine-tuning. Our future work will focus on the construction of a dynamic model by integrating data from metabolomics analysis and including additional microorganisms identified in other industrial farms following similar fermentation protocols.

## Conclusion

Our work reveal a core of microorganism shared between farms of industrial relevance. This is a crucial step to rational design starter cultures, reducing the fermentation times, and controlling the expression of undesirable phenotypes. Similar approaches could be applied to other farms of industrial relevance to further dissect fine-flavour cocoa fermentation at different locations. Moreover, our genome-scale community model is the first report of a community metabolic model for industrial fermentations of fine-flavour cocoa. Consequently, it represents a valuable platform to further study species-dependent reactions (Supplementary File S1), which could be further improved by including the reactions of new microorganisms and additional metabolomic/fluxomic data.

## Experimental section

### Cocoa beans fermentation

Cocoa beans used in this work were originated from a standard mixture of fine-flavour clones (i.e. FTA-2, FSA-13, FSV-41, Fear-5, ICS-39, ICS-95, EET-8, CAUC 37-39, TSH 565 and Luker40) harvested at two different large-scale producing farms in Colombia.

For further information regarding the selected clones, please refer to the International Cocoa Germplasm Database (Available at http://www.icgd.reading.ac.uk).

The selected industrial farms: Necoclí farm (8°28′48.24″N 76°40′59.86*″*W) and Arauca farm (7°01′01*″*N 71°25′01*″*W) are located at two different agro-ecological zones, which are separated by the Andes Mountains (approximately 610 km from each other).

During the main harvest season of 2018 (October–November), two industrial large-scale pre-standardized fermentations were performed at the selected farms. Cocoa pods were harvested, piled-up on the ground, and manually opened with a machete. All pods were harvested the same day. The cocoa pulp-bean mass was then scooped out from the pods by hand and collected into plastic barrels (load capacity ~ 50 kg). Once filled, barrels were immediately transferred to a customized fermentation room with pre-arranged wooden boxes. Two wooden boxes for fermentation were arranged at each farm. In Necoclí farm, 400 kg of cocoa pulp-bean mass were weighed and placed into each box. In the case of Arauca farm, a total of 350 kg of cocoa pulp-bean mass were weighed and transferred to fermentation boxes. Cocoa sweatings were drained away over fermentation through holes at the bottom of the boxes. The cocoa pulp-bean mass was mixed every 48 h to allow oxygenation using an iron shovel. All fermentations were completed after six days. Fermentation quality was confirmed by cut test evaluation of cocoa beans (ISO standard 2451:2017)^60,61^. It is important to mention, that these fermentation conditions are the standardized procedures always conducted at these farms.

### Fermentation mass sampling

Samples of 10 beans were collected every 24 h starting at day 0 (i.e. just after placing the cocoa pulp-bean mass into each box) until the end of fermentation (day 6). Beans were collected from different points in the fermentation mass (e.g. surface and bottom) to ensure representative samples. On each farm, a total of six-replicates was collected every day, four of them from fermentation box 1 and the remaining two from fermentation box 2. Each sample was ground using a sanitized coffee grinder and subsequently stored at 4 °C in Nucleic Acid Preservation (NAP) Buffer^62^ until DNA extraction.

### DNA extraction from fermentation samples

The extraction of DNA from fermentation samples was performed according to the protocol described by Ha et al.^63^. Briefly, DNA was extracted using DNA extraction buffer (Cetyl trimethyl ammonium bromide CTAB/Proteinase K) and precipitated on CTAB/NaCl. Then, ethanol was used to ensure the complete removal of CTAB, and the resulting DNA was finally eluted in Milli-Q water and checked for quality.

### Library preparation and Illumina sequencing

Two amplicon libraries were prepared, using V3-V4 hypervariable regions for bacterial *16S* rRNA gene, and the internal transcribed spacer ITS1-ITS5 for fungi. PCR reactions were performed in 25 μL containing 5X Hot FirePol Blend Master Mix (SOLIS BIODYNE), primers (Supplementary Table S1) at 0.4 μM, and 2 μL of DNA. PCRs were performed on BIORAD C100 thermal cyclers under the following conditions: initial denaturation at 95 °C for 5 min, followed by 35–40 cycles of 30 s at 95 °C, 30 s at the primer annealing temperature (Supplementary Table S1) and 1 min at 72 °C, and final extension for 10 min. For bacterial *16S* amplification, the primers used already contain barcodes and Illumina adapters as previously reported^64,65^. For the ITS amplicons, Illumina adapters were ligated using the TruSeq Nano HT kit. Samples were purified using AMPure XP beads (BECKMAN COULTER) and normalized with the SequalPrep Normalization Plate Kit (APPLIED BIOSYSTEMS) for a final pooling into two libraries (*16S* and ITS respectively). DNA sequencing was carried out on Illumina MiSeq sequencer (ILLUMINA, San Diego, CA, USA), PE 2 × 250 using 500 cycle V2 MISEQ Reagent Kits and standard flow cells on an Illumina MiSeq platform located at the laboratory of CorpoGen Corporation (Bogotá, Colombia).

### Taxonomic profiling

The paired-end reads generated by Illumina MiSeq were initially merged using the overlapping sequence information. Primer sequences and adapters were subsequently removed to obtain trimmed reads. To filter out those sequences with the low quality a quality filtering was performed using the EzBioCloud^66^ quality-filtering tool for bacteria, the FASTQC v0.11.9 (http://www.bioinformatics.babraham.ac.uk/projects/fastqc) tool, and ARB-SILVA^67^ for fungi. Thus, all sequences with lengths of < 100 bp or > 2,000 bp, averaged Q values lower than 30, and not predicted as *16S* gene or ITS, were removed. Non-redundant reads were removed, and identical sequences were de-replicated to reduce processing time. These sequences were subsequently used to determine the taxonomic profile for each sample. In all cases, species rarefaction curves were completely saturated. The prokaryotic EzBioCloud *16S* database (PKSSU4.0)^68^ and the UNITE fungal ITS database (https://unite.ut.ee/)^69^ were used to find and calculate sequence similarities using the VSEARCH program^70^ for bacteria and fungi, respectively. Different sequence similarity cut-offs were used for taxa identification as reported by Yarza et al.^71^, where 97% is taken as a cut-off for species-level identification. Chimeric sequences were detected and removed applying UCHIME^72^ algorithms to trusted non-chimeric reference databases.

### Microbial analysis

The Shannon diversity index was calculated per each sample using the previously obtained OTUs (operational taxonomic units) information as reported^73^. Selected microorganisms sharing metabolic and physiological traits were clustered in four groups: Lactic Acid Bacteria, Acetic Acid Bacteria, Yeasts, and Molds. Clustering was performed based on the taxonomic profiling previously described, where selected genera were clustered according to their current classification: ALB^31^, AAB^74^, Yeasts, and Molds^75^. The relative abundance of these four groups of microorganisms was recorded every 24 h during fermentation starting at day 0 (i.e. just after placing the cocoa pulp-bean mass into each box) until the end of fermentation (day 6). Besides, the relative abundance of selected dominant bacterial and fungal species was also tracked over the fermentation. Dominant species were defined as species detected all over the replicates and showing a relative abundance higher than 1% from the total number of bacterial or fungal species.

### Reconstruction of a genome-scale metabolic community model

The genome-scale metabolic models of *Saccharomyces cerevisiae*, *Bacillus subtilis* subsp. subtilis str. 168, *Limosilactobacillus fermentum* IFO 3956 and *Acetobacter aceti* were used to reconstruct a genome-scale metabolic community model for fine flavour cocoa fermentation. These microorganisms have been selected for the community model as they were identified as dominant species shared between the studied farms. Two of these models (i.e. *Saccharomyces cerevisiae* model iMM904^47^ and *Bacillus subtilis* subsp. *subtilis* str. 168 iYO844 model^48^) have been previously reported, while metabolic models for *Limosilactobacillus fermentum* and *Acetobacter aceti* were reconstructed from *Limosilactobacillus fermentum* str. IFO 3956 genome (GCF_000010145.1)^51^ and *Acetobacter aceti* str. TMW2.1153 genome (GCF_002005445.1)^52^, respectively. De novo metabolic models were reconstructed using the PATRIC database^53,54^ with Reconstruction Metabolic Model Services. A completed media parameter was chosen for metabolic reconstructions. Flux Balance Analysis-FBA^55^ was performed for each of the four metabolic models. The SteadyCom optimization framework^56^ was used to reconstruct a community metabolic model. The *createCommModel()* function was applied to integrate the four independent metabolic models into a unique community model by giving them a different set of identifiers for each organism, which resembles a model with separate compartments. Minor corrections were made to the community model to fix reaction directions. No changes for uptake systems were made. ESCHER tool^76^ was used to generate metabolic maps and further edited using Inkscape software (0.92.5 version).

### Cocoa liquor production and sensory evaluation

The fermented cocoa beans at each farm were sun-dried, roasted, and converted into cocoa liquor by CASALUKER S.A (Bogotá, Colombia) following their standardized protocols. Briefly, cocoa liquor is obtained by subsequent steps of cleaning (i.e. Nibs are separated from shells), roasting, cooling, winnowing, grinding, tempering, molding, and storage for further analysis. Details on liquor production are protected as casaluker´s intellectual property. Sensory evaluation of cocoa liquor was performed by a trained tasting panel of 6 members of CASALUKER S.A. This a regular standardized procedure performed several times a year at the company using liquor produced with material arriving from these farms. Flavour descriptors included acid/sour, astringent, cocoa, bitter, fruity, floral, nutty, moldy, green, malt and others. The analysis was performed in triplicate with a temperature of evaluation between 45–50 °C. The flavour intensity was expressed as numerical values ranging from 0 to 9.

## Supplementary Information

Supplementary Information 1.

Supplementary Information 2.

## Data Availability

All data generated or analyzed during this study are included in this published article (and its Supplementary Information files).
