# Supplementary material for: Dissecting industrial fermentations of fine flavour cocoa through metagenomic analysis
Source: Sci Rep. 2021 Apr 21;11:8638. doi: 10.1038/s41598-021-88048-3 (PMC8060343; doi:10.1038/s41598-021-88048-3)
Supplement: Supplementary file 1 — Supplementary Information 1. [file 41598_2021_88048_MOESM1_ESM.pdf]

DISSECTING INDUSTRIAL FERMENTATIONS OF FINE FLAVOUR COCOA THROUGH METAGENOMIC ANALYSIS

Miguel Fernández-Niño, María Juliana Rodríguez-Cubillos, Fabio Herrera-Rocha, Juan Manuel Anzola, Martha Lucia Cepeda-Hernández, Jenny Lorena Aguirre Mejía, María José Chica, Héctor Hugo Olarte, Claudia Rodríguez-López, Dayana Calderón, Adan Ramírez-Rojas, Patricia Del Portillo, Silvia Restrepo, Andrés Fernando González Barrios.

SUPPLEMENTARY INFORMATION

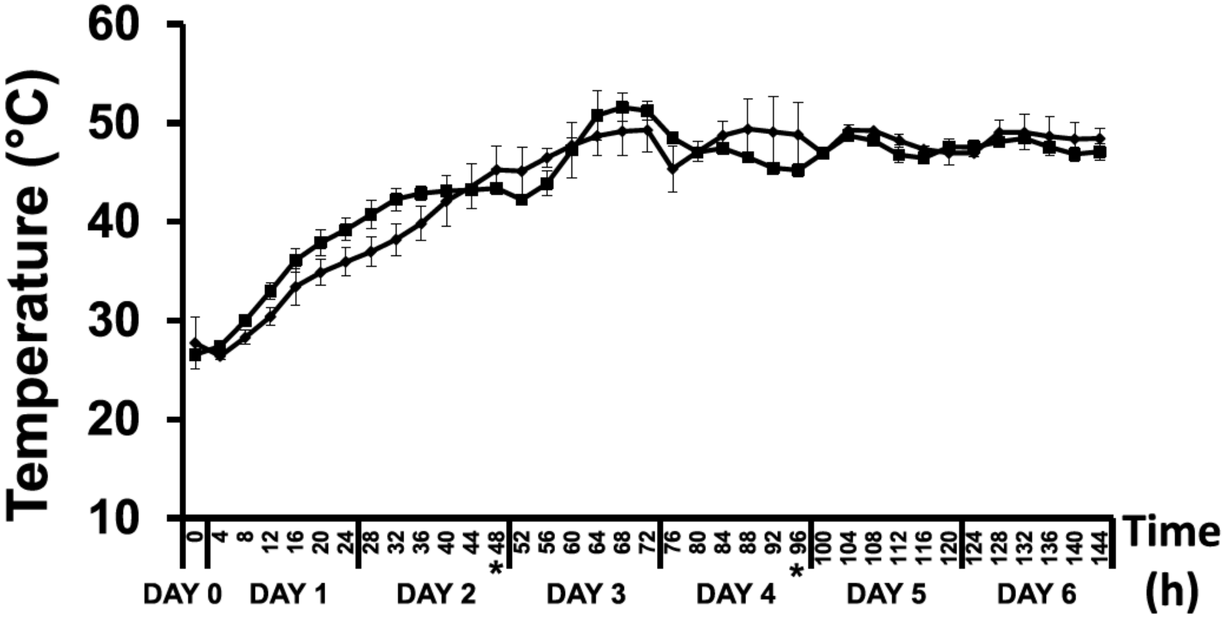

Supplementary Figure S1. Evolution of temperature during cocoa fermentation at two farms owned by CasaLuker S.A: Temperature is always measured for each fermentation performed by the company using temperature sensors located at the center of the fermenting mass. Error bars indicate standard deviation of three replicates. The cocoa pulp-bean mass is mixed every 48 hours to allow oxygenation as indicated by asterisks. Data is shown for Arauca (■) and Necoclí (●) farms.

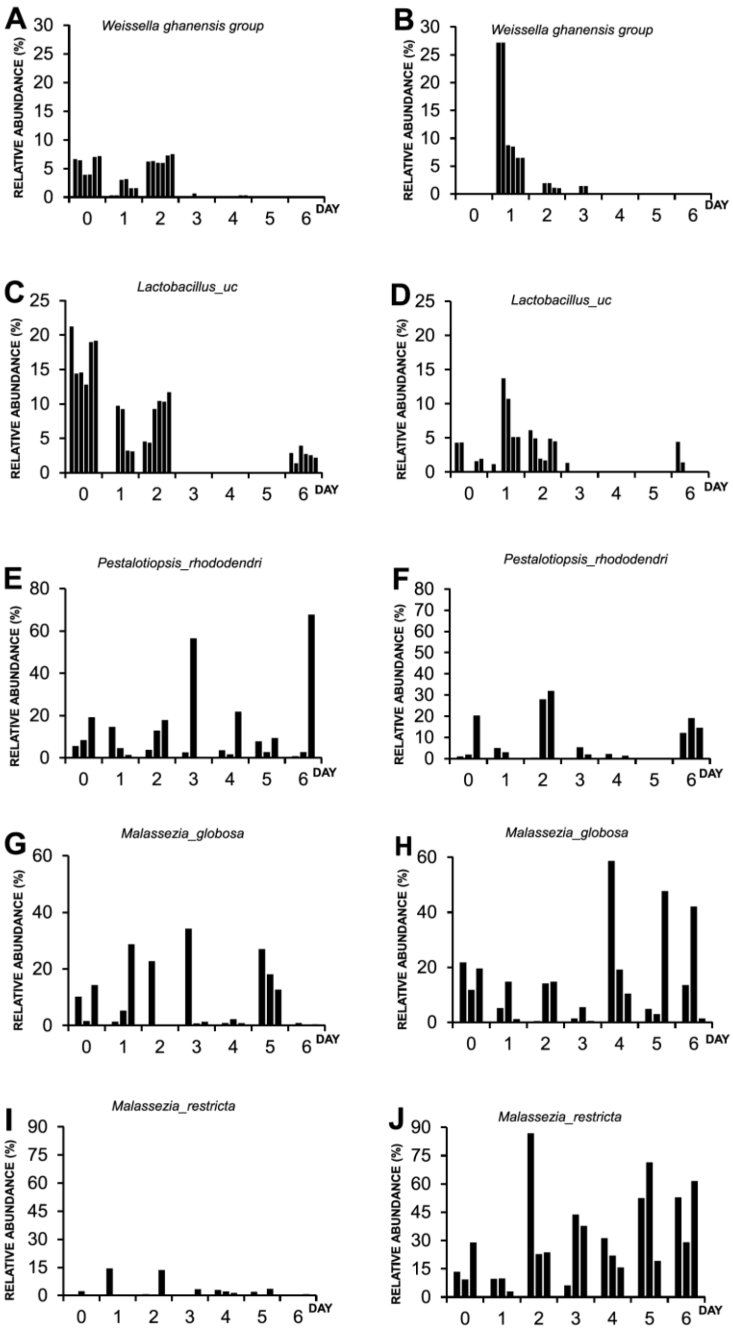

Supplementary Figure S2. Relative abundance of dominant species/groups of bacteria and fungi from the microorganism's core during the fermentation of fine-flavour cocoa beans at Necoclí (A, C, E, G, and I) and Arauca (B, D, F, and J) farms. Data is shown for species not included in the metabolic model. Dominant species were defined as species detected all over the replicates and showing a relative abundance higher than 1% from the total number of bacterial or fungi species. Each bar represents a replicate. At least two replicates per day have been analyzed.

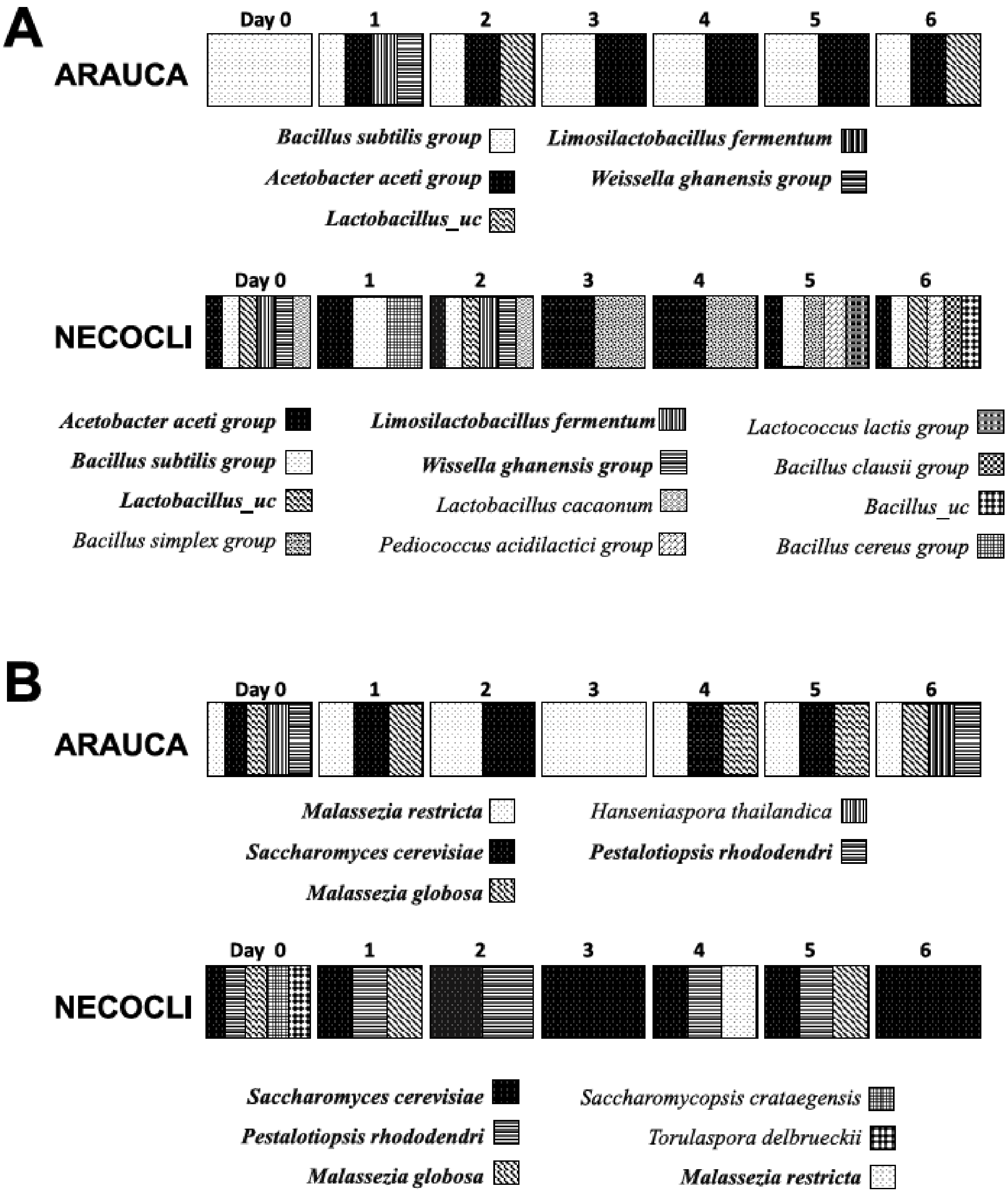

Supplementary Figure S3. Dominant species/groups of bacteria (A) and fungi (B) identified every day during the fermentation of fine-flavour cocoa beans at Arauca and Necoclí farms. Block patterns indicate the presence/absence of a certain dominant species but not abundance. Dominant species were defined as species detected all over the replicates and showing a relative abundance higher than 1% from the total number of bacterial or fungi species.

Supplementary Table S1. Primer sequences used in this study.

Primer name	Sequence (5'-3')	Amplicon size (bp)	Annealing temperature (°C)	Ref.
16S_V3-F	CCTACGGGAGGCAGCAG	250-300	55	Kozich *et al* (2013)
16_V4-R	GGACTACHVGGGTWTCTAAT			
ITS_1-F	CAAGAGATCCGTTGTTGAAAGTK	120-400	55	Epp *et al* (2012)
ITS_5-R	GGAAGTAAAAGTCGTAACAAGG			
